# Long non-coding RNA FER1L4 inhibits prostate cancer progression via sponging miR-92a-3p and upregulation of FBXW7

**DOI:** 10.1186/s12935-020-1143-0

**Published:** 2020-02-28

**Authors:** Wei Huo, Fei Qi, Kaichen Wang

**Affiliations:** 1 Department of Urology, China-Japan Union Hospital, Jilin University, 126 Xiantai Street, Changchun, 130001 People’s Republic of China; 2 Department of Operating Room, China-Japan Union Hospital, Jilin University, Changchun, 130001 People’s Republic of China

**Keywords:** FER1L4, FBXW7, miR-92a-3p, Prostate cancer, YAP1 signaling

## Abstract

**Background:**

Dysregulation of long non-coding RNAs (lncRNAs) is involved in development of prostate cancer. However, the molecular mechanisms of many lncRNAs in prostate cancer have not been studied yet.

**Methods:**

The lncRNA Fer-1-like protein 4 (FER1L4) expression was explored in prostate tumors and normal prostate tissues by RT-qPCR and bioinformatic analysis. Overexpression of FER1L4 was performed to evaluate its role in prostate cancer cell proliferation and survival. The molecular mechanism of FER1L4 was investigated by dual luciferase reporter assay, RNA pull down assay, western blotting and RT-qPCR.

**Results:**

It was found that FER1L4 was lower in prostate cancer tissues than normal tissues. Higher expression of FER1L4 was associated with prostate cancer tissues of early stage (AJCC stage I/II). Overexpression of FER1L4 inhibited cell proliferation and promoted cell apoptosis in prostate cancer cells. Bioinformatic analysis, RT-qPCR, RNA pull down assay and dual luciferase assay showed that FER1L4 upregulated F-box/WD repeat-containing protein 7 (FBXW7) tumor suppressor via sponging miR-92a-3p. Silencing of FBXW7 reversed the cell phenotypes caused by FER1L4 overexpression in prostate cancer cells.

**Conclusion:**

The data demonstrated that FER1L4, a downregulated lncRNA in prostate cancer, was pivotal for cell proliferation and survival of prostate cancer. The study provided new sights into understanding of the signaling network in prostate cancer and implied that FER1L4 might be a biomarker for patients with prostate cancer.

## Background

Prostate cancer is the second most commonly diagnosed cancer type for males, accounting for approximately 13.5% of newly diagnosed cancer cases in 2018 [1]. A total of 359,000 patients died from prostate cancer annually worldwide [1]. The prognosis of patients with prostate cancer has been greatly improved with the development of system therapeutic approach including androgen deprivation, chemotherapy and surgery [2, 3]. However, the emergence of castration resistance and chemotherapy resistance limits the efficacy of current treatment and threatened patients’ lives [4, 5]. Therefore, discovery of novel biomarkers and investigation of molecular mechanisms of prostate cancer may provide insights for the diagnosis and treatment of prostate cancer.

Long non-coding RNAs (lncRNAs) are a class of endogenous non-coding RNAs with more than 200 nucleotides in length [6]. Studies showed that lncRNAs could bind to RNA, DNA or protein to exert their biological functions [7, 8]. According to competing endogenous RNA (ceRNA) hypothesis [9], lncRNAs sponged miRNAs to compete their binding to target gene mRNA and regulated gene expression. Recent years, multiple studies revealed that lncRNA were implicated in cancer pathogenesis and progression [10–12]. High throughout sequencing demonstrated that there were numerous differentially expressed lncRNAs between prostate tumors and normal tissues [13]. Many lncRNAs were identified as oncogenes or tumor suppressors in prostate cancer [14, 15]. For example, lncRNA HOXD-AS1 was highly expressed in castration-resistant prostate cancer and inhibited cell proliferation and chemotherapy resistance via recruiting WDR5 [14]. LncRNA NEAT1 facilitated oncogene transcription by epigenetic modification of gene promoter in PC-3 and VAaP cells [15]. LncRNA MEG3 sponged miR-9-5p, upregulated QKI-5 and suppressed prostate cancer cell proliferation, migration, invasion and induced apoptosis [16]. Fer-1-like protein 4 (FER1L4) have recently attracted the researchers’ attention due to its involvement in the progression of cancer [17, 18]. The biological role and molecular mechanism of FER1L4 in prostate cancer is unknown.

F-box/WD repeat-containing protein 7 (Fbxw7) is frequently mutated in human cancers of many types [19]. As a well-known F-box protein, FBXW7 is a component of E3 ligase complex, mediating the recognizing and binding of complex to specific target proteins [20]. Via targeting oncogenes for degradation, FBXW7 functioned as a tumor suppressor to attenuate uncontrolled cell proliferation and induced cell apoptosis in cancer cells [21–24]. In hepatocellular carcinoma, FBXW7 promoted cell apoptosis and ceased cell growth through targeting YAP1 for degradation [21]. In several cancer types, downregulation of FBXW7 was responsible for elevation of c-Myc and cancer progression [22–24]. FBXW7 also played a tumor suppressor role in prostate cancer cells [25].

The current study aimed to investigate the role of FER1L4 in prostate cancer. The expression of FER1L4 was detected in prostate tumors and matched normal tissues. The function role of FER1L4 was explored in prostate cancer cells by cell proliferation and cell apoptosis assays. Bioinformatic analysis, RNA pull down assay, western blotting and dual luciferase reporter assay were applied to study the molecular mechanism of FER1L4 in prostate cancer cells.

## Materials and methods

### Research subjects

A total of 78 prostate tumors and adjacent normal tissues were obtained from patients with prostate cancer during surgical removal of tumors in China-Japan Union hospital during July, 2015 to September, 2018. All patients did not receive chemotherapy or radiotherapy before surgery. The patients were aged 45–67 with a median age of 57.6 ± 6.6. The tumors were staged as stage I (11 cases), II (23 cases), III (27 cases) and IV (17 cases) prostate tumors according to the American Joint Committee on Cancer (AJCC) staging system [26]. The present study was approved by Institutional Ethics Review Board of China-Japan Union hospital. Written informed consents were obtained from all participants. The tissues were stored in – 80 ℃ before subjected to RNA extraction.

### Cell culture

Human prostate cancer cell lines PC-3, LNCaP, DU145 and normal prostate cell line RWPE-1 were purchased from American Type Culture Collection (ATCC, Manassas, VA). These cells were maintained in DMEM (Invitrogen, Carlsbad, CA) supplemented with 10% FBS (Gibco; Invitrogen) and 1% penicillin/streptomycin solution (Invitrogen) in a 37℃ incubator with 5% CO_2_.

### Overexpression of FER1L4 and silencing of FBXW7

Full length of FER1L4 was amplified from PC-3 cDNA by Taq DNA Polymerase (Thermo Fisher Scientific) and ligated into pcDNA3 plasmid (YouBio, Changsha, China). Empty pcDNA3 or pcDNA3-FER1L4 was transfected into PC-3 and DU145 cells with the Lipofectamine 3000 reagent (Invitrogen) following producer’s protocol. Control siRNA (5′-UUCUCCGAACGUGUCACGUTT-3′) and FBXW7 siRNA (5′-GUGAAGUUGUUGGAGUAGATT-3′) were purchased from GenePharma (Shanghai, China). To silence FBXW7 expression, FBXW7 siRNA or control siRNA was transfected into PC-3 and DU145 cells with the Lipofectamine RNAiMax reagent (Invitrogen) following producer’s protocol. 48 h after transfection, the transfection efficiency was detected by RT-qPCR or western blotting.

### Elevation and inhibition of miR-92a-3p

miR-NC mimic (5′-UUCUCCGAACGUGUCACGU-3′), miR-NC inhibitor (5′-UUGUCCGAACGUGUCACGU-3′), miR-92a-3p mimic (5′-UAUUGCACUUGUCCCGGCCUGU-3′) and miR-92a-3p inhibitor (5′-ACAGGCCGGGACAAGUGCAAUA-3′) were synthesized by RiboBio (Guangzhou, China). miR-92a-3p inhibitor is single-stranded, modified RNA which can tightly bind to endogenous miR-92a-3p and effectively downregulate miR-92a-3p in cells. For transfection, 20 nM miR-NC mimic or miR-NC inhibitor or miR-92a-3p mimic or miR-92a-3p inhibitor was mixed with Lipofectamine 3000 in Opti-MEM and added into the cells in each well of 24-well plate. 48 h after transfection, the transfection efficiency was detected by RT-qPCR.

### RNA extraction and RT-qPCR

Total RNA was extracted from cells and tissues with TRIzol reagent (Invitrogen) following manufacturer’s protocol. RNA was reversed transcribed into first-stranded cDNA with the RevertAid RT Reverse Transcription Kit (Thermo Fisher Scientific, Waltham, MA). RT-qPCR was performed with TB Green Fast qPCR Mix (TaKaRa, Tokyo, Japan) on a CFX96 Touch Real-time PCR Detection System (Bio-Rad). The relative gene expression was calculated by the 2^−ΔΔCt^ method [27]. U6 and β-actin were internal controls for miRNA and mRNA/lncRNA respectively. The primer sequences were listed in Table 1.

**Table 1:** Primer sequences

Primer name	Sequence
FER1L4-forward	5′-CCGTGTTGAGGTGCTGTTC-3′
FER1L4-reverse	5′-CCCATCCCAGGAGGTCACCT-3′
FBXW7-forward	5′-CGACGCCGAATTACATCTGTC-3′
FBXW7-reverse	5′-CGTTGAAACTGGGGTTCTATCA-3′
YAP1-forward	5′-TAGCCCTGCGTAGCCAGTTA-3′
YAP1-reverse	5′-TCATGCTTAGTCCACTGTCTGT-3′
CTGF-forward	5′-ACCGACTGGAAGACACGTTTG-3′
CTGF-reverse	5′-CCAGGTCAGCTTCGCAAGG-3′
CYR61-forward	5′-ACCGCTCTGAAGGGGATCT-3′
CYR61-reverse	5′-ACTGATGTTTACAGTTGGGCTG-3′
β-actin-forward	5′-CATGTACGTTGCTATCCAGGC-3′
β-actin-reverse	5′-CTCCTTAATGTCACGCACGAT-3′
Stem-loop	5′-CTCAACTGGTGTCGTGGAGTCGGCAATTCAGTTGAGACAGGC-3′
miR-92a-3p-forward	5′-GCCGAGTATTGCACTTGTCC-3′
miR-92a-3p-reverse	5′-CTCAACTGGTGTCGTGGA-3′
U6-forward	5′-TGAGAACTGAATTCCATGGGTT-3′
U6-reverse	5′-ACGCTTCACGAATTTGCGT-3′

### Protein extraction and western blotting

Bcl-2 (#15071, 1:2000), Bax2 (#5023, 1:2000), AKT (#4691, 1:2000), p-AKT (#4060, 1:2000) and YAP1 (#14074, 1:2000) antibodies were bought from Cell Signaling Technology (Danvers, MA). FBXW7 (#AB36334, 1:2000) antibody was obtained from AbSci (College Park, MD). β-actin antibody (#SAB1305567, 1:5000) was purchased from Sigma Aldrich (Darmstadt, Germany). HRP-conjugated secondary antibodies against rabbit (#ABL3012-2, 1:10,000) and mouse (#ABL3031-2, 1:10,000) were products of AbSci. Lysates were prepared with the RIPA lysis buffer (Thermo Fisher Scientific) following manufacturer’s protocol. The concentration of lysates was determined with the BCA Protein Assay Kit (Thermo Fisher Scientific). 20 μg proteins were loaded in the 8% SDS-PAGE gel and transferred onto a PVDF membrane. The membrane was blocked in 5% non-fat milk at room temperature for 1 h. After that, the membrane was incubated with primary antibodies and secondary antibodies sequentially at room temperature for 1 h. The membrane was developed with the ECL Western Blotting Substrate (Pierce; Thermo Fisher Scientific). The intensity of bands was quantified with the ImageJ v.1.5.1 (National Institute of Health).

### Protein stability assay

The stability of YAP1 protein was detected by the MG132 assay. 48 h after transfection of vectors and siRNAs, cells were treated with either 20 μM MG132 (Selleck, Houston, TX) or equal amount of DMSO for additional 12 h. After that, cells were harvested, subjected to protein extraction and western blotting.

### Bioinformatic analysis

The potential target miRNA of FER1L4 was predicted on the RegRNA 2.0 software (http://regrna2.mbc.nctu.edu.tw/). The binding site of miR-92a-3p on the 3′UTR of FBXW7 was predicted by TargetScan software (http://www.targetscan.org/vert_72/) and miRanda (http://www.microrna.org/microrna/home.do). The expression of FER1L4 and miR-92a-3p in TCGA dataset was analyzed by ENCORI (http://starbase.sysu.edu.cn/). GEPIA (http://gepia.cancer-pku.cn/index.html) was used to analyze correlation between expression of FER1L4 and FBXW7 by Pearson correlation analysis.

### Detection of caspase-3 activity analysis

The caspase-3 activity was detected with an EnzCheck^®^ Caspase-3 Assay kit#1 (Invitrogen) following manufacturer’s protocol. Briefly, 2 × 10^5^ cells were lysed in 50 µL cell lysis buffer provided in the kit on ice for 30 min, followed by centrifugation (4000*g*, 5 min). After that, 50 µL substrate working solution (the mixture of Z-DEVD-AMC substrate and reaction buffer) was added to the supernatant and maintained at room temperature for 30 min. The fluorescent intensity at a wavelength of 405 nm was determined by the Gemini XPS fluorescent plate reader (Sunnyvale, CA).

### Cell proliferation and cell apoptosis assay

The proliferation ability of cells was determined with a CCK-8 kit (DoJinDo, Tokyo, Japan). In a brief, 10 μL CCK-8 solution was added to each well of the plate and sustained for 1 h at 37 ℃. The absorbance at 450 nM was detected by a Microplate Reader (Bio-Rad) to reflect cell number. The apoptotic cells were detected by a Dead Cell Apoptosis Kit with Annexin V FITC and PI, for flow cytometry (Invitrogen). Harvested cells were suspended in Annexin binding buffer provided by the kit and stained with Annexin V-FITC and PI at room temperature for 15 min. After that, cells were subjected to the flow cytometry analysis on a MACSQuant X (Miltenyi, Bergisch Gladbach, Germany). The data was analyzed by the FlowJo software. Annexin V+/PI+ and Annexin V+/PI− cells were apoptotic cells.

### Dual luciferase reporter assay

FER1L4 was subcloned from pcDNA3-FER1L4 to pGL3 plasmid (Promega, Madison, WI). 3′UTR of FBXW7 was amplified from PC-3 cDNA and ligated into pGL3 plasmid. Point site mutations were introduced into pGL3-FER1L4 and pGL3-FBXW7 with the Quick Site-Directed Mutation Kit (Agilent; Thermo Fisher Scientific). pGL3-FER1L4-WT, pGL3-FBXW7-WT, pGL3-FER1L4-Mut and pGL3-FBXW7-Mut was co-transfected with pRL-TK (Promega) into PC-3 and DU145 cells and sustained for 48 h. After that, the luciferase activity of each well was detected with a Dual-Luciferase^®^ Reporter Assay System (Promega). The firefly luciferase was normalized to renilla luciferase.

### RNA pull down assay

The interaction between miR-92a-3p and FER1L4 was studied via an RNA pull down assay. Biotin labeled miR-92a-3p wild type (Biotin-miR-92a-3p-WT) and miR-92a-3p mutant (Biotin-miR-92a-3p-Mut) were synthesize and purchased from RiboBio (Guangzhou, China). In a brief, 50 nM Biotin-miR-92a-3p-WT or Biotin-miR-92a-3p-Mut was transfected into PC-3 cells. After 48 h, the cells were lysed in lysis buffer and the lysates were incubated with M-280 streptavidin magnetic beads (Sigma-Aldrich) pre-treated with RNase-free BSA and yeast tRNA (TRNABAK-RO; Sigma-Aldrich) at 4 ℃ for 3 h. The lysates were then washed with lysis buffer, low salt buffer and high salt buffer sequentially. The bound RNAs were extracted by TRIzol reagent and the expression of FER1L4 was detected by the RT-qPCR.

### Statistical analysis

The data were analyzed with GraphPad Prism 6.0 software and presented as mean ± SD. The correlation between miR-92a-3p expression and FER1L4 expression was analyzed by the Pearson Correlation analysis. Two groups were compared with Student’s t test. Three groups were compared with one-way ANOVA followed by Newman Keuls analysis. p < 0.05 was considered as statistically significant.

## Results

### Low expression of FER1L4 is observed in prostate cancer

**Fig. 1:**
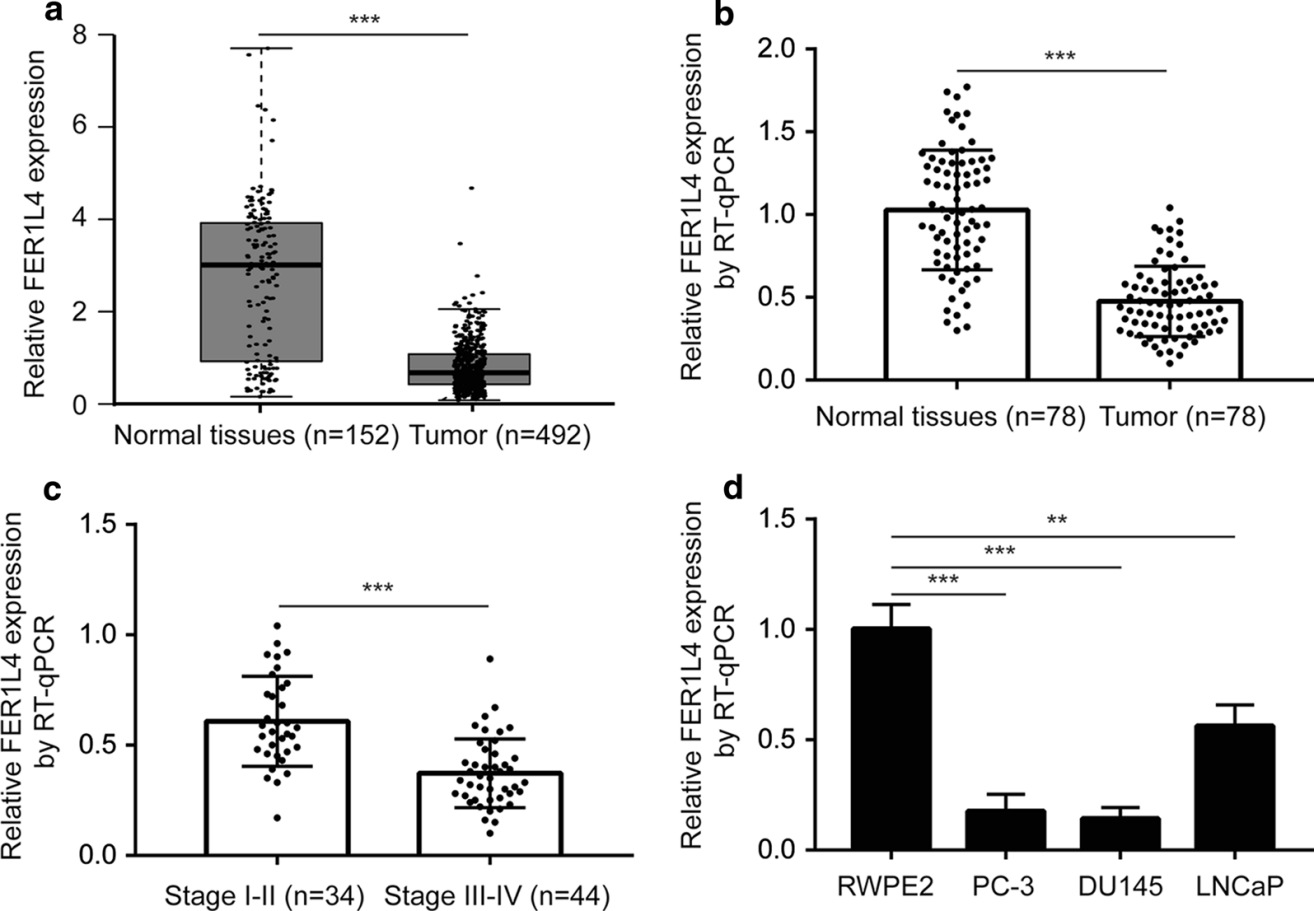
FER1L4 was downregulated in prostate cancer. **a** Analysis of TCGA-PRAD data showed that FER1L4 was downregulated in prostate cancer tissues (n = 492) compared with normal prostate tissues (n = 152). **b** RT-qPCR was performed to investigate the expression of FER1L4 in our collected prostate cancer tissues (n = 78) compared with matched normal prostate tissues (n = 78). The results showed that FER1L4 was decreased in tumors. **c** Relatively lower expression of FER1L4 was observed in later stage prostate cancer tissues (III–IV) compared with those of early stage (I–II). **d** RT-qPCR was performed to detect FER1L4 expression in a panel of prostate cancer cell lines and the immortal normal prostate cells. It was showed that FER1L4 was downregulated in prostate cancer cell lines (PC-3, DU145, LNCaP) compared with the immortal normal prostate cell line (RWPE2). **p < 0.01; ***p < 0.001

We firstly analyzed FER1L4 expression in TCGA-PRAD dataset. FER1L4 was significantly decreased in prostate cancer tissues (n = 492) compared with normal prostate tissues (n = 152) (Fig. 1a). In our collected specimens, RT-qPCR also showed that FER1L4 was downregulated in prostate cancer tissues (n = 78) compared with matched normal prostate tissues (n = 78) (Fig. 1b). In addition, lower expression of FER1L4 was detected in tumors of later stage (Stage III–IV, n = 44) compared with those of early stage (Stage I–II, n-34) (Fig. 1c). Moreover, we found that FER1L4 expression was decreased in prostate cancer cells (PC-3, DU145, LNCaP) compared with the immortalized human prostatic epithelial cells (RWPE2) (Fig. 1d).

### Overexpression of FER1L4 induces cell apoptosis and suppress cell proliferation in prostate cancer cells

**Fig. 2:**
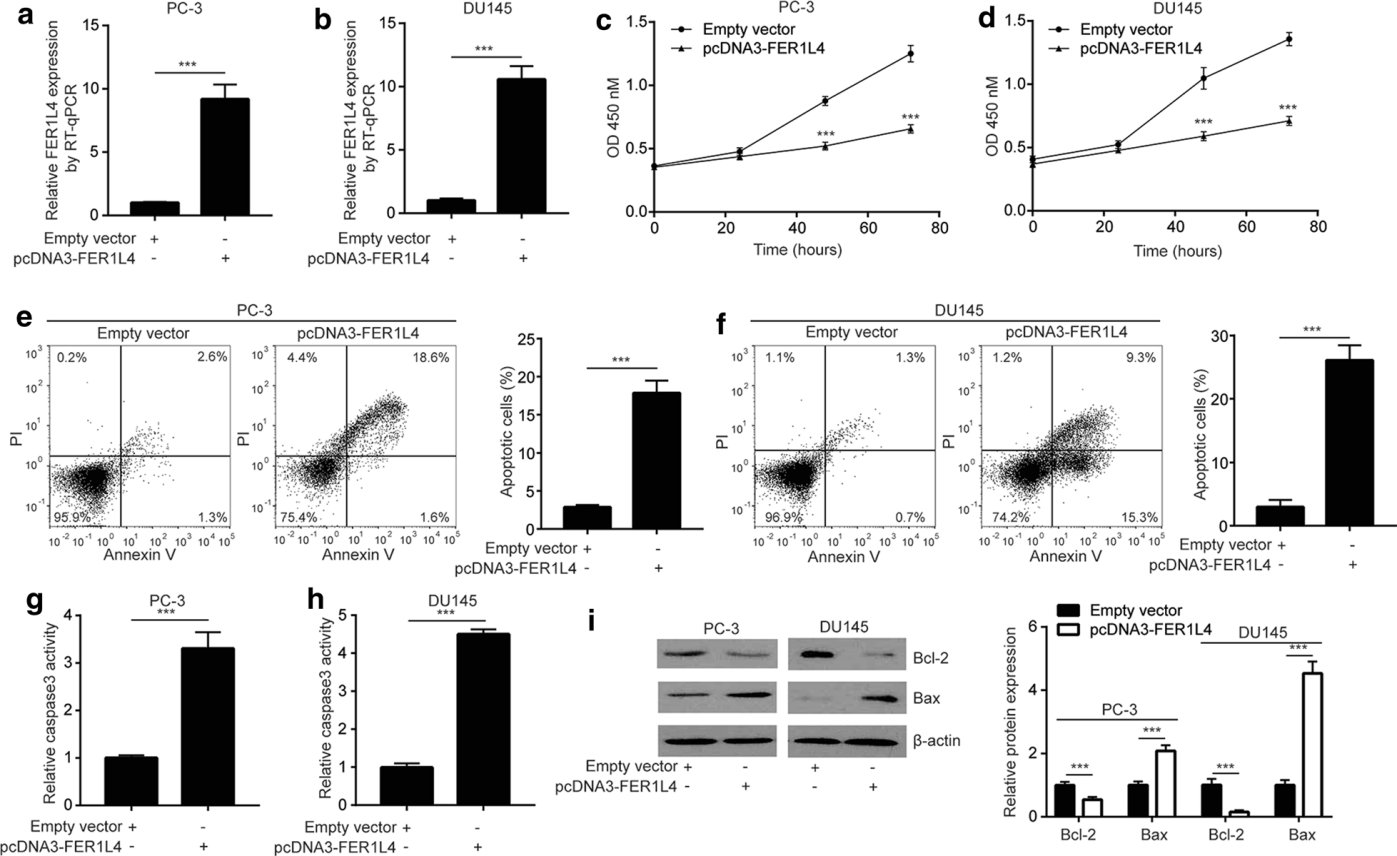
Overexpression of FER1L4 inhibited cell proliferation and induced cell apoptosis in prostate cancer cells. **a**, **b** RT-qPCR was performed to detect the transfection efficiency of pcDNA3-FER1L4 in PC-3 and DU145 cells. Transfection of pcDNA3-FER1L4 elevated FER1L4 expression in PC-3 (**a**) and DU145 (**b**) cells. **c**, **d** The CCK-8 assay was used to detect the cell proliferation ability. Overexpression of FER1L4 decreased absorbance at OD 450 nM, indicating inhibition of cell proliferation in PC-3 (**c**) and DU145 (**d**) cells. **e**, **f** Flow cytometry was used to analyze the percentage of apoptotic cells. Overexpression of FER1L4 increased percentage of cells positive for PI staining, indicating induction of apoptosis in PC-3 (**e**) and DU145 (**f**) cells. **g**, **h** Overexpression of FER1L4 increased caspase3 activity in PC-3 (**g**) and DU145 cells (**h**). **i** Western blotting showed that overexpression of FER1L4 decreased Bcl-2 and increased Bax protein expression in PC-3 and DU145 cells. ***p < 0.001

We overexpressed FER1L4 in prostate cancer cells by transfection of pcDNA3-FER1L4. Transfection of pcDNA3-FER1L4 induced a tenfold elevation of FER1L4 in PC-3 and DU145 cells (Fig. 2a, b). Elevation of FER1L4 greatly suppressed cell proliferation in PC-3 and DU145 cells (Fig. 2c, d). Cell apoptosis might contribute to decreased cell proliferative ability. The cell apoptosis assay showed that FER1L4 overexpression evoked cell apoptosis in PC-3 and DU145 cells (Fig. 2e, f). Consistent with observation in the cell apoptosis assay, it was found that the caspase-3 activity was significantly elevated upon FER1L4 overexpression (Fig. 2g, h). The expression of Bcl2, the anti-apoptotic protein, was decreased whereas Bax, the pro-apoptotic protein, was increased in PC-3 and DU145 cells (Fig. 2i).

### FER1L4 sponges miR-92a-3p in prostate cancer cells

**Fig. 3:**
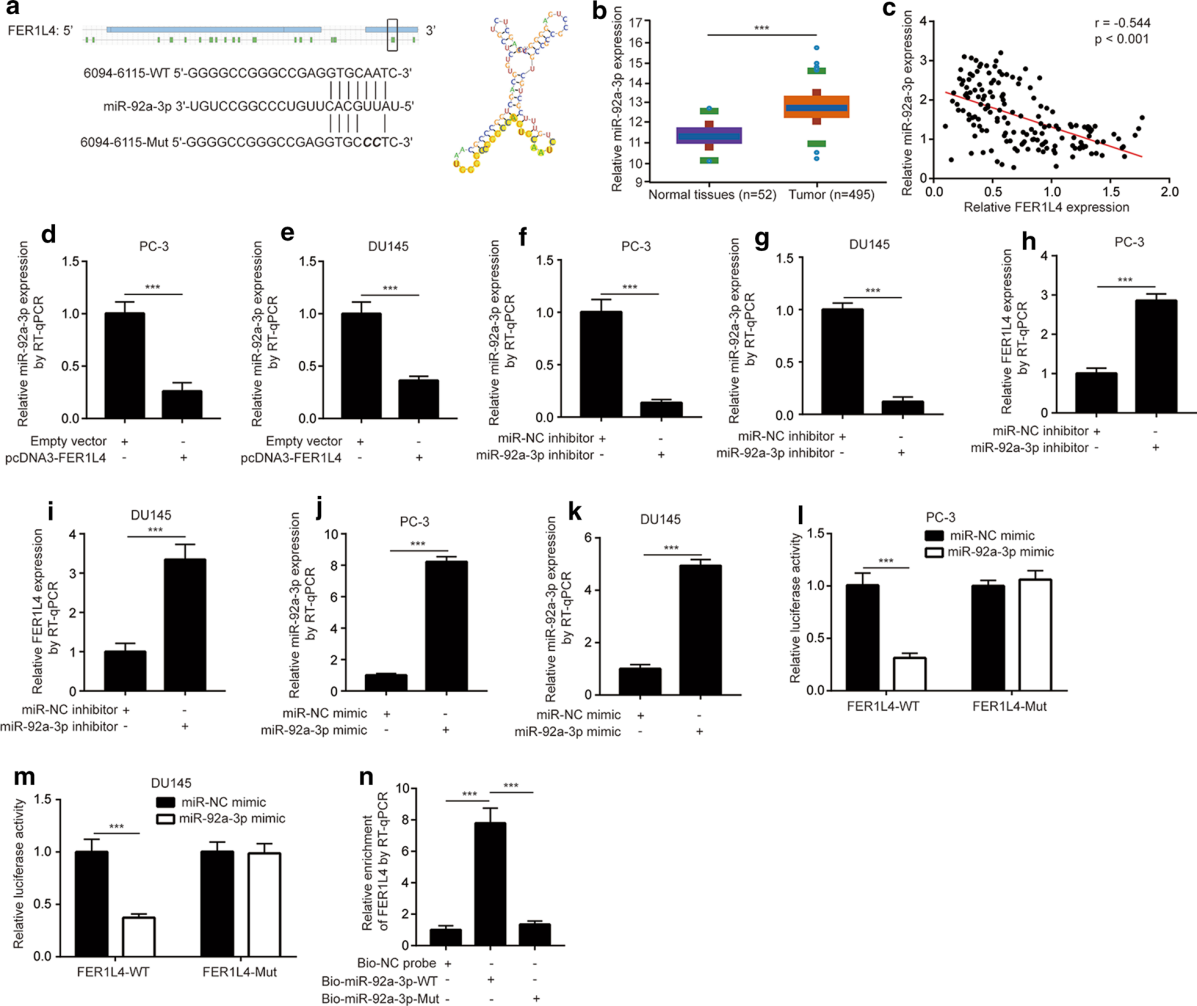
FER1L4 sponged miR-92a-3p in prostate cancer cells. **a** RegRNA 2.0 software predicted the potential binding site and secondary structure of FER1L4/miR-92a-3p interaction. **b** Analysis of TCGA-PRAD showed that miR-92a-3p was overexpressed in prostate cancer tissues (n = 495) compared with normal prostate tissues (n = 52). **c** RT-qPCR was applied to detect miR-92a-3p expression in 78 pairs of tumors and normal tissues. Pearson correlation analysis indicated a strong negative correlation between miR-92a-3p and FER1L4 expression. **d**, **e** RT-qPCR showed that overexpression of FER1L4 decreased miR-92a-3p expression in PC-3 (**d**) and DU145 (**e**) cells. **f**, **g** RT-qPCR showed that transfection of miR-92a-3p inhibitor decreased miR-92a-3p expression in PC-3 (**f**) and DU145 (**g**) cells. H-I. RT-qPCR showed that decreased expression of miR-92a-3p elevated FER1L4 expression in PC-3 (**h**) and DU145 cells (**i**). **j**, **k** RT-qPCR showed that transfection of miR-92a-3p mimic increased miR-92a-3p expression in PC-3 (**j**) and DU145 cells (**k**). **l**, **m** The dual luciferase reporter assay was applied to analyze the direct binding between miR-92a-3p and FER1L4. Overexpression of miR-92a-3p reduced relative luciferase activity of pGL3-FER1L4-WT in PC-3 (**l**) and DU145 cells (**m**). **n** In the RNA pull down assay, RT-qPCR was performed to analyze the enrichment of FERL14 by Bio-NC probe, Bio-miR-92a-3p-WT and Bio-miR-92a-3p-Mut. ***p < 0.001

Studies showed that FER1L4 functioned as a ceRNA to regulate gene expression and disease progression [18, 28]. Using RegRNA 2.0, miR-92a-3p was predicted as a potential binding miRNA for FER1L4 with the highest score. The secondary structure of miR-29a-3p and FER1L4 interaction and the complementary binding sites between miR-29a-3p and FER1L4 were presented in Fig. 3a. It was found that miR-92a-3p facilitated prostate cancer cell proliferation [29]. In addition, in contrast to low expression of FER1L4, the analysis of TCGA-PRAD dataset showed that miR-92a-3p was significantly upregulated in prostate cancer tissues (n = 495) compared with normal tissues (n = 52) (Fig. 3b), indicating a potential regulatory association between miR-92a-3p and FER1L4. Moreover, in our collected tumors and normal samples, there was a strong negative correlation (r = -0.544) between FER1L4 and miR-92a-3p expression (Fig. 3c). In PC-3 and DU145 cells, overexpression of FER1L4 decreased miR-92a-3p levels (Fig. 3d, e). We next downregulated miR-92a-3p in prostate cancer cells by transfection of miR-92a-3p inhibitor. MiR-92a-3p inhibitor decreased miR-92a-3p in PC-3 and DU145 cells (Fig. 3f, g). Downregulation of miR-92a-3p increased FER1L4 expression in PC-3 and DU145 cells (Fig. 3h, i). We then used dual luciferase reporter assay to confirm their direct regulatory association. MiR-92a-3p mimic was transfected into PC-3 and DU145 to upregulate miR-92a-3p expression (Fig. 3j, k). In the dual luciferase assay, we found that miR-92a-3p overexpression decreased relative luciferase activity of pGL3-FER1L4 in PC-3 cells (Fig. 3l), which was also observed in DU145 cells (Fig. 3m). To investigate the direct interaction between miR-92a-3p and FER1L4, we performed RNA pull down assay. The results suggested that FER1L4 enrichment was significantly increased by Bio-miR-92a-3p-WT in PC-3 cells (Fig. 3n).

### FBXW7 mRNA is directly targeted by miR-92a-3p

**Fig. 4:**
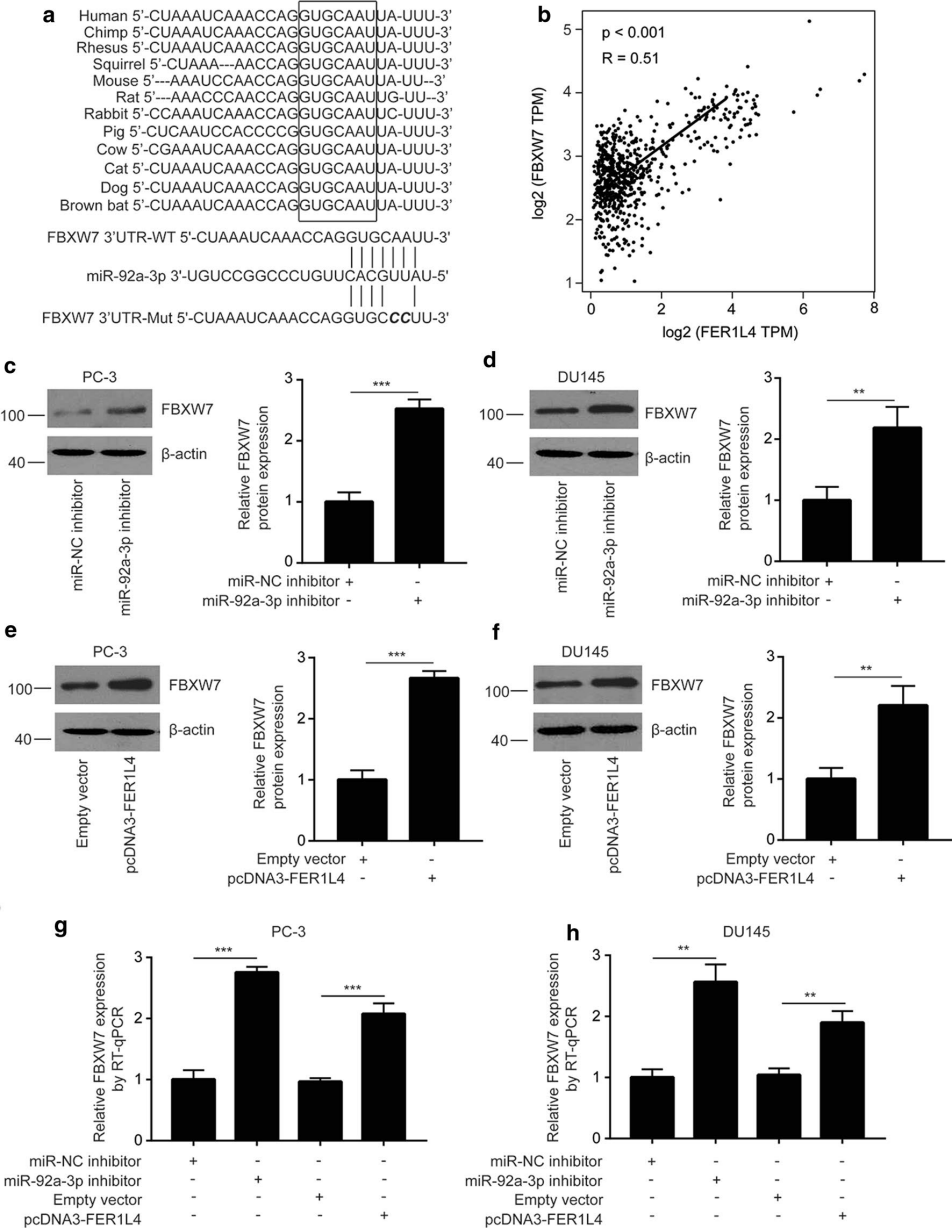
FBXW7 was a target gene of miR-92a-3p in prostate cancer. **a** Using TargetScan, sequencing alignment showed that there was a conserved binding site for miR-92a-3p on the 3′UTR of FBXW7 mRNA. **b** With GEPIA software, bioinformatic analysis indicated that there was a strong positive correlation between FBXW7 expression and FER1L4 expression in prostate cancer tissues. **c**, **d** Western blotting showed that inhibition of miR-92a-3p elevated FBXW7 protein expression in PC-3 (**c**) and DU145 cells (**d**). **e**, **f** Western blotting showed that overexpression of FER1L4 increased FBXW7 protein expression in PC-3 (**e**) and DU145 cells (**f**). **g**, **h** RT-qPCR showed that inhibition of miR-92a-3p or overexpression of FER1L4 increased FBXW7 mRNA expression in PC-3 (**g**) and DU145 cells (**h**). **p < 0.01; ***p < 0.001

**Fig. 5:**
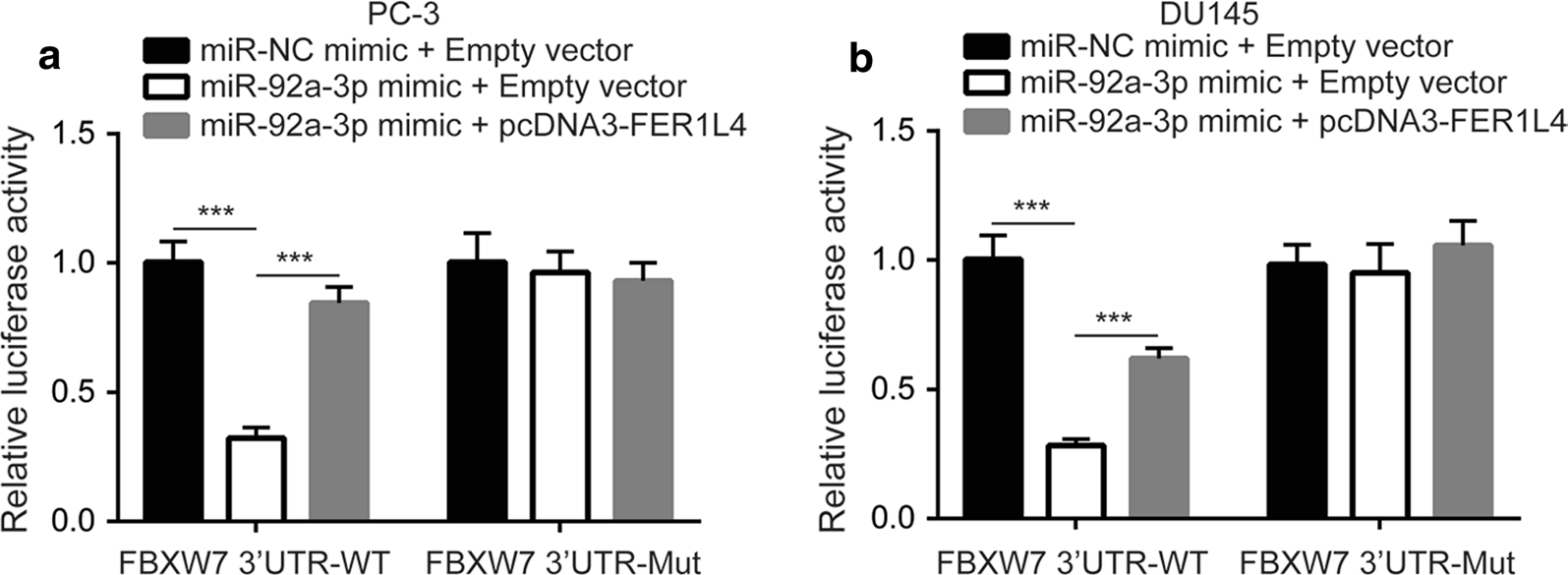
MiR-92a-3p directly bound to 3′UTR of FBXW7 mRNA in prostate cancer cells. **a**, **b** In the dual luciferase reporter assay, miR-92a-3p overexpression reduced relative luciferase activity of pGL3-FBXW7 3′UTR-WT which was reversed upon FBXW7 overexpression in PC-3 (**a**) and DU145 cells (**b**). ***p < 0.001

We used miRanda and TargetScan softwares to predict target genes of miR-92a-3p. Among thousands of predicted target genes of miR-92a-3p, FBXW7, a tumor suppressor, was one of the top five precited target genes of miR-92a-3p in both softwares. The potential binding site for miR-92a-3p on mRNA of FBXW7 was conserved among species (Fig. 4a). Moreover, there was a strong positive correlation between FBXW7 expression and FER1L4 levels in TCGA-PRAD (p < 0.001, R = 0.51) (Fig. 4b). Western blotting showed that inhibition of miR-92a-3p elevated FBXW7 protein expression in PC-3 cells (Fig. 4c) and DU145 cells (Fig. 4d). Similarly, overexpression of FER1L4 increased FBXW7 protein expression in PC-3 cells (Fig. 4e) and DU145 cells (Fig. 4f). RT-qPCR showed that miR-92a-3p inhibition and FER1L4 overexpression elevated FBXW7 mRNA expression in PC-3 (Fig. 4g) and DU145 cells (Fig. 4h). To validate the direct association, we used dual luciferase reporter assay. Overexpression of miR-92a-3p reduced relative luciferase activity of FBXW7 3′UTR-WT which was reversed after FER1L4 overexpression in PC-3 cells (Fig. 5a). The similar results were observed in DU145 cells (Fig. 5b). These data collectively indicated a FER1L4/miR-92a-3p/FBXW7 axis in prostate cancer.

### The FER1L4/miR-92a-3p/FBXW7 axis determines the activity of YAP1 signaling in prostate cancer

**Fig. 6:**
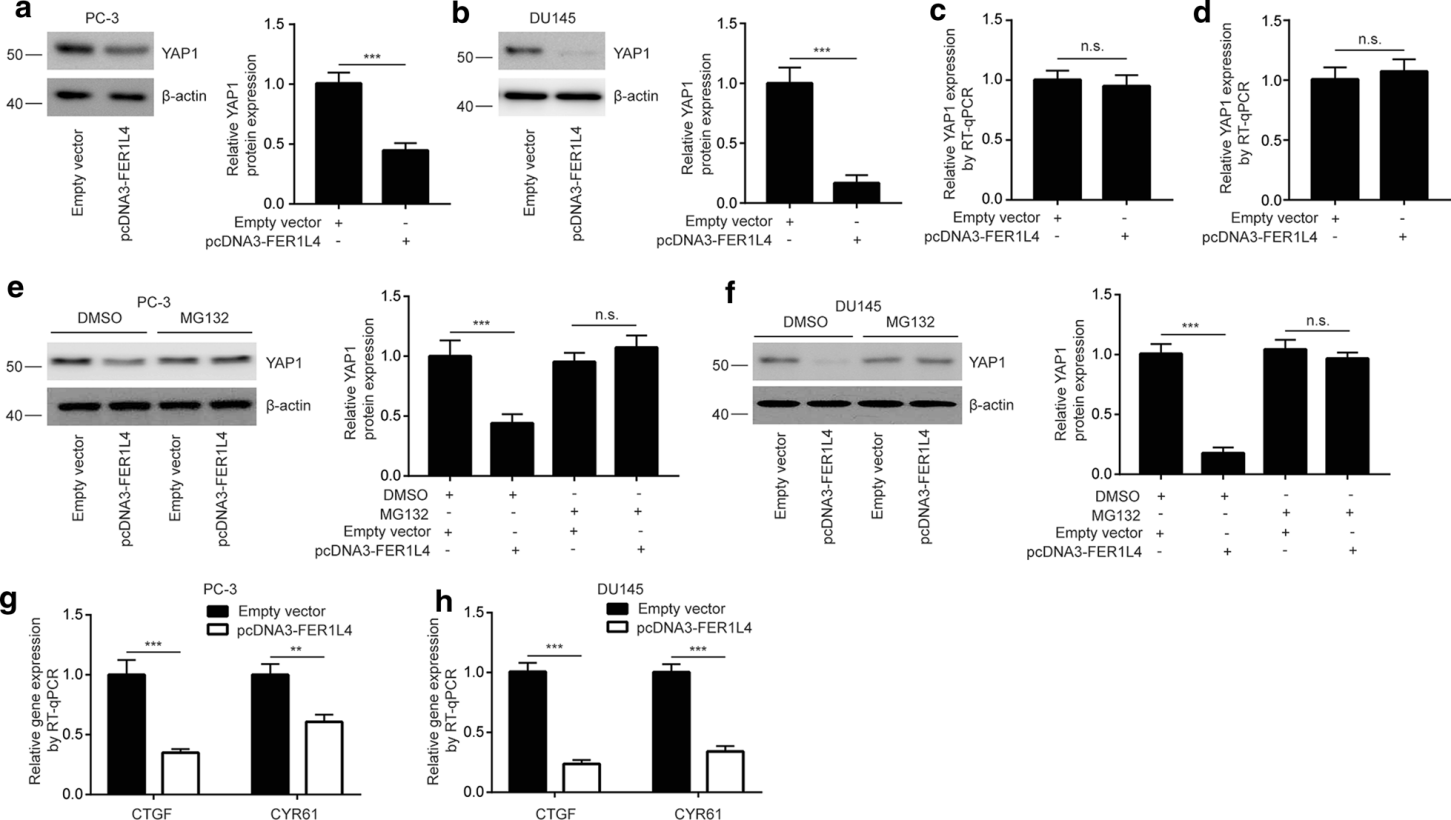
YAP1 expression was repressed by the FER1L4/miR-92a-3p/FBXW7 axis in prostate cancer cells. **a**, **b** Western blotting showed that overexpression of FER1L4 decreased YAP1 protein expression in PC-3 (**a**) and DU145 cells (**b**). **c**, **d** RT-qPCR showed that overexpression of FER1L4 did not altered YAP1 mRNA expression in PC-3 (**c**) and DU145 cells (**d**). **e**, **f** MG132 treatment reversed downregulation of YAP1 upon FER1L4 overexpression in PC-3 (**e**) and DU145 cells (**f**). **g**, **h** The target gene of YAP1 signaling (CTGF, CYR61) was downregulated in PC-3 (**g**) and DU145 (**h**) cells transfected with FER1L4. **p < 0.01; ***p < 0.001

As a member of E3 complex, FBXW7 exerted its tumor suppressor function via targeting oncogenes (such as YAP1) for degradation. As we expected, FER1L4 overexpression decreased YAP1 protein expression in PC-3 (around 50%) and DU145 cells (around 80%), the difference may be due to different cell backgrounds (Fig. 6a, b). The mRNA levels of YAP1 was not altered towards FER1L4 overexpression (Fig. 6c, d). In the protein stability assay, the expression of YAP1 was sustainable in the presence of MG132, a specific 26-S proteasome inhibitor, in PC-3 cells transfected with FER1L4 (Fig. 6e), indicating the decreased YAP1 protein expression was due to instability of protein. The similar results were observed in DU145 cells (Fig. 6f). Furthermore, the mRNA levels of YAP1 target genes (CTGF, CYR61) were reduced after FER1L4 overexpression in PC-3 and DU145 cells (Fig. 6g, h). The data demonstrated that the FER1L4/miR-92a-3p/FBXW7 axis controlled the key signaling pathway in prostate cancer cells.

### FER1L4 suppresses cell proliferation and induce cell apoptosis via upregulation of FBXW7 in prostate cancer cells

**Fig. 7:**
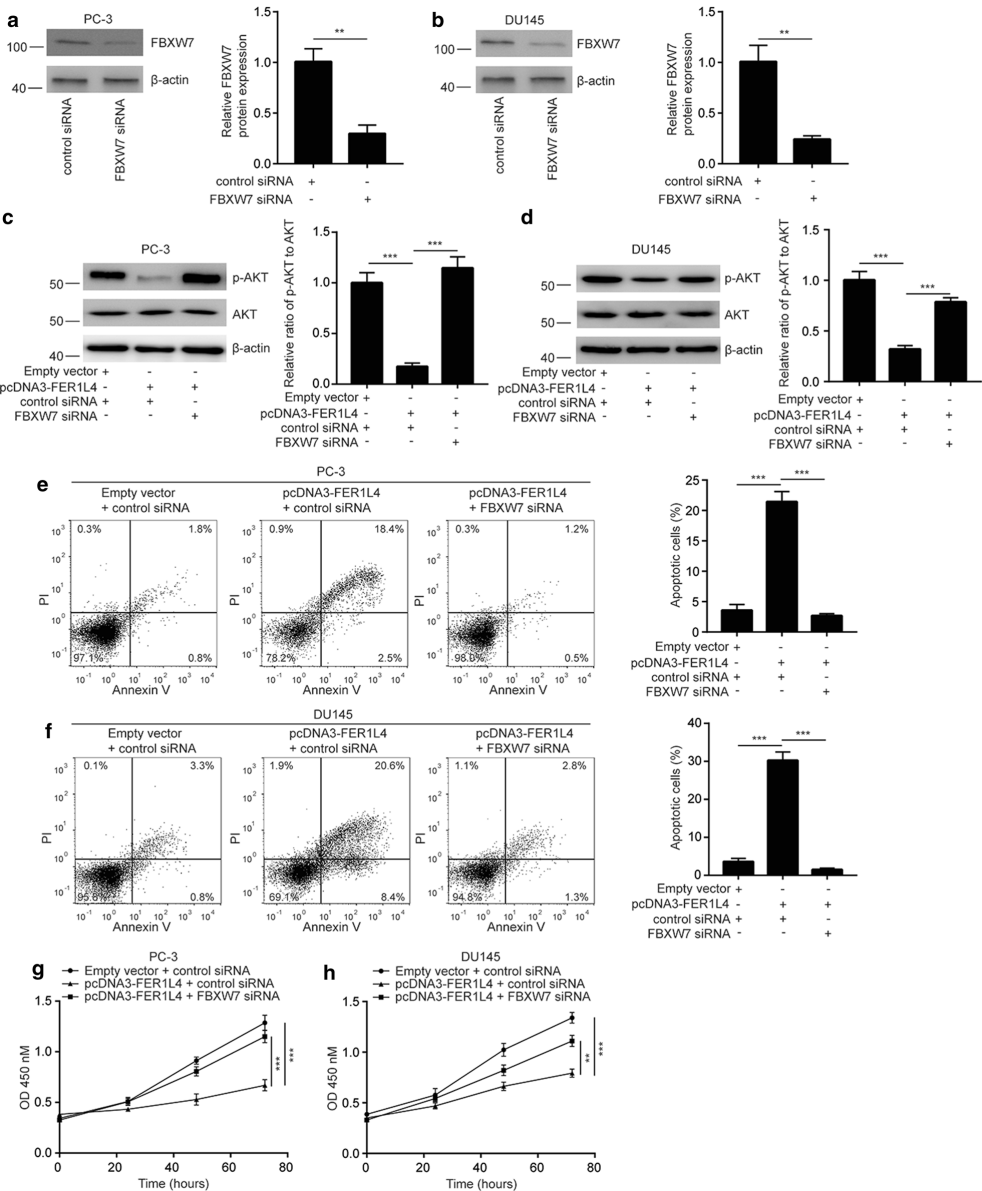
FER1L4 regulated cell proliferation and apoptosis via controlling FBXW7 expression in prostate cancer cells. **a**, **b** Transfection of FBXW7 siRNA decreased FBXW7 protein expression in PC-3 (**a**) and DU145 cells (**b**). **c**, **d** Overexpression of FER1L4 decreased the ratio of p-AKT/AKT protein which was reversed after FBXW7 silencing in PC-3 (**c**) and DU145 cells (**d**). **e**, **f** FBXW7 silencing attenuated cell apoptosis induced by FER1L4 overexpression in PC-3 (**e**) and DU145 cells (**f**). **g**, **h** FBXW7 silencing attenuated cell proliferation inhibition induced by FER1L4 overexpression in PC-3 (**g**) and DU145 cells (**h**). **p < 0.01; ***p < 0.001

**Fig. 8:**
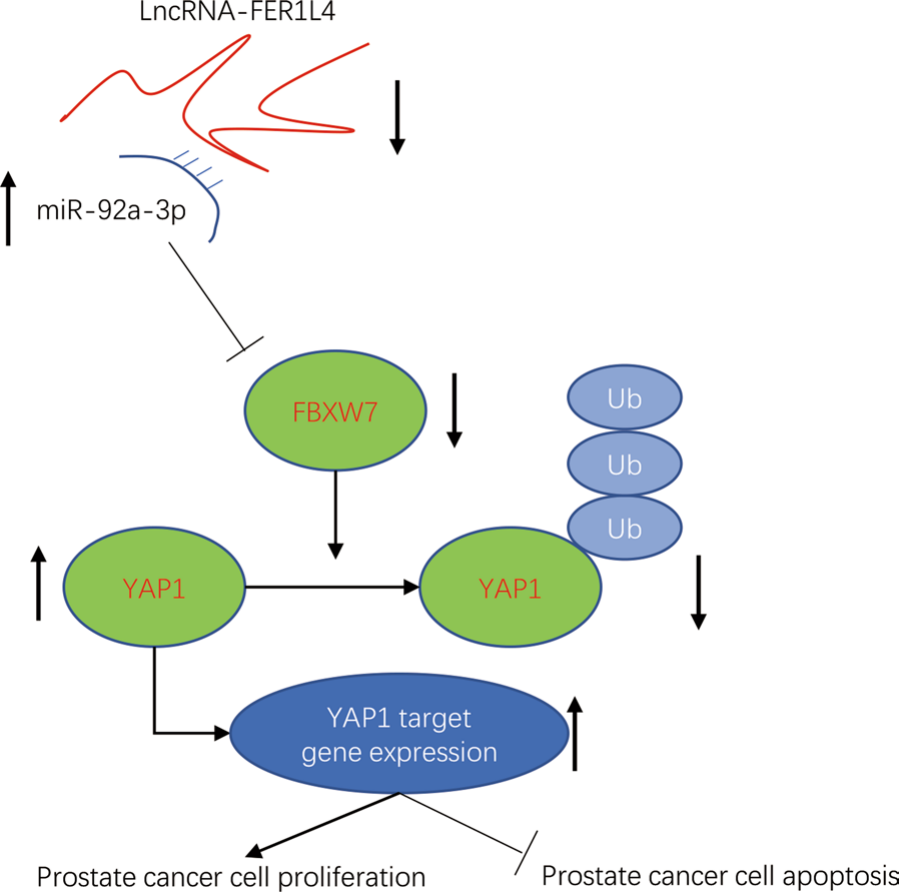
A proposed model for FER1L4/miR-92a-3p/FBXW7 axis in prostate cancer cells

We silenced FBXW7 expression in PC-3 and DU145 by transfection of FBXW7 siRNA (Fig. 7a, b). The activity of PI3K/AKT pathway was critical for survival and proliferation of prostate cancer and tightly regulated by FER1L4 in several cancer types [30, 31]. Interestingly, in PC-3 and DU145 cells, we also observed that FER1L4 overexpression decreased the phosphorylation of AKT protein which was reversed towards FBXW7 silencing (Fig. 7c, d), indicating PI3K/AKT signaling was also regulated by FER1L4/FBXW7 in prostate cancer cells. Additionally, FBXW7 silencing reversed FER1L4 overexpression induced cell apoptosis in PC-3 and DU145 cells (Fig. 7e, f). In addition, FBXW7 silencing attenuated cell proliferation inhibition induced by FER1L4 overexpression in PC-3 and DU145 cells (Fig. 7g, h). The results revealed that FER1L4 control prostate cancer proliferation and apoptosis via upregulation of FBXW7 and downregulation of YAP1 and its target gene expression (Fig. 8).

## Discussion

Numerous studies showed that lncRNAs were highly involved in initiation, development, drug resistant and metastasis of prostate cancer [32, 33]. Song et al. initially identified lncRNA-FER1L4 as one of most significantly downregulated lncRNAs in gastric cancer via bioinformatic analysis of lncRNA microarray data [34]. Later, several studies suggested that FER1L4 could function as an oncogene or a tumor suppressor in different cancer types [18, 35–37]. The most well-characterized role of FER1L4 is its tumor suppressor function as a negative regulator of AKT signaling in cancers [30, 31]. Many studies showed that FER1L4 inactivated AKT signaling to suppress cancer progression including osteosarcoma, lung cancer, hepatocellular carcinoma and endometrial carcinoma [28, 38, 39]. FER1L4 also repressed esophageal squamous cell carcinoma proliferation [37]. In glioma, however, FER1L4 promoted cancer progression via sponging miR-371 and upregulation of E2F1 [18]. In the present study, we firstly studied the role of FER1L4 in prostate cancer. Similar to its role in most other cancer types, FER1L4 was also downregulated in prostate cancer. Transfection of FER1L4 further revealed that FER1L4 inhibited cell proliferation and induced cell apoptosis in prostate cancer cells. The current findings introduced a pivotal role of FER1L4 in prostate cancer.

The progression of prostate cancer is driven by dysregulation of miRNAs [40, 41]. MiR-92a-3p was overexpressed in several cancer types and was involved in cancer development [42–44]. MiR-92a-3p was a highly expressed miRNA in exosomes from prostate cancer cells [45]. In the present study, our bioinformatic analysis suggested that miR-92a-3p was also elevated in prostate cancer tissues. MiR-92a-3p could cooperate with other three miRNAs to downregulate PTEN and promoted cell proliferation in prostate cancer [29]. FBXW7 was downregulated and suppressed several cancer progression including prostate cancer [21, 23, 25]. The downregulation of FBXW7 was due to gene mutation and aberrant expression of several miRNAs [46]. MiR-25-3p directly suppressed FBXW7 to facilitate glioma cell proliferation [47]. FBXW7 was also targeted by miR-223 in oral squamous cell carcinoma [48]. miR-92a-3p targeted FBXW7 to promote cell proliferation and invasion in cervical cancer [49]. We confirmed the direct association between FBXW7 and miR-92a-3p in prostate cancer cells.

Studies on FER1L4 suggested that FER1L4 mainly exerted its function via sponging miRNAs in cancer cells [18, 28]. We predicted that FER1L4 sequence harbored binding site for miR-92a-3p. Their mutual regulatory association and direct interaction were verified via RT-qPCR, RNA pull down and dual luciferase reporter assays. Additionally, the expression of FBXW7 was positively correlated with FER1L4 in prostate cancer tissues. Thus, the current study revealed a FER1L4/miR-92a-3p/FBXW7 axis in prostate cancer.

YAP1 signaling is pivotal for cancer cell proliferation, metastasis and resistance to cell apoptosis [50]. Hyperactivation of YAP1 signaling was reported in prostate cancer due to epigenetic modification and altered expression of non-coding RNAs [51, 52]. FBXW7 was responsible for degradation of YAP1, thereby controlling cell proliferation and apoptosis [21]. As a positive regulator of FBXW7, we found that FER1L4 decreased stability of YAP1 protein to reduce its expression in prostate cancer cells. Overexpression of FER1L4 also reduced mRNA expression of YAP1 target genes. Our work provided new understandings for the regulation of YAP1 signaling by FER1L4/miR-92a-3p/FBXW7 axis in prostate cancer.

## Conclusion

In conclusion, the study demonstrated a FER1L4/miR-92a-3p/FBXW7 axis in prostate cancer. FER1L4 might be a biomarker and therapeutic target for patients with prostate cancer.

## Data Availability

They are available under special request.
